# Anthropogenic N Deposition Slows Decay by Favoring Bacterial Metabolism: Insights from Metagenomic Analyses

**DOI:** 10.3389/fmicb.2016.00259

**Published:** 2016-03-02

**Authors:** Zachary B. Freedman, Rima A. Upchurch, Donald R. Zak, Lauren C. Cline

**Affiliations:** ^1^School of Natural Resources and Environment, University of MichiganAnn Arbor, MI, USA; ^2^Department of Ecology, Evolution, and Behavior, University of MichiganAnn Arbor, MI, USA

**Keywords:** metagenome, saprotroph, N deposition, C storage, climate change

## Abstract

Litter decomposition is an enzymatically-complex process that is mediated by a diverse assemblage of saprophytic microorganisms. It is a globally important biogeochemical process that can be suppressed by anthropogenic N deposition. In a northern hardwood forest ecosystem located in Michigan, USA, 20 years of experimentally increased atmospheric N deposition has reduced forest floor decay and increased soil C storage. Here, we paired extracellular enzyme assays with shotgun metagenomics to assess if anthropogenic N deposition has altered the functional potential of microbial communities inhabiting decaying forest floor. Experimental N deposition significantly reduced the activity of extracellular enzymes mediating plant cell wall decay, which occurred concurrently with changes in the relative abundance of metagenomic functional gene pathways mediating the metabolism of carbohydrates, aromatic compounds, as well as microbial respiration. Moreover, experimental N deposition increased the relative abundance of 50 of the 60 gene pathways, the majority of which were associated with saprotrophic bacteria. Conversely, the relative abundance and composition of fungal genes mediating the metabolism of plant litter was not affected by experimental N deposition. Future rates of atmospheric N deposition have favored saprotrophic soil bacteria, whereas the metabolic potential of saprotrophic fungi appears resilient to this agent of environmental change. Results presented here provide evidence that changes in the functional capacity of saprotrophic soil microorganisms mediate how anthropogenic N deposition increases C storage in soil.

## Introduction

Changes in the composition and function of microbial communities can amplify or moderate anthropogenic modification of global biogeochemical cycles (Falkowski et al., 2008; Zhao et al., 2014). However, our ability to mechanistically link changes in microbial community composition with metabolic responses that control biogeochemical cycles has been limited, especially in regard to the molecular mechanisms slowing plant litter decay under future rates of atmospheric N deposition (Zak et al., 2008; Liu and Greaver, 2010; Edwards et al., 2011; Eisenlord et al., 2013; Freedman and Zak, 2014). By the end of this century, terrestrial ecosystems in the eastern United States and Europe will receive quantities of anthropogenic nitrogen (N) that are unprecedented over the history of life on Earth (Galloway et al., 2004; Torseth et al., 2012). This aspect of global change has the potential to constrain the accumulation of anthropogenic CO_2_ in the Earth's atmosphere (Magnani et al., 2007; Zak et al., 2008), and therefore, slow the pace of climate warming. For 20 years, we have conducted a long-term field experiment that simulates future rates of anthropogenic N deposition to understand microbial processes mediating ecosystem C storage. Our experiment has revealed that future rates of anthropogenic N deposition have reduced microbial decay, which substantially increased organic matter accumulation in forest floor (+51%) and surface mineral soil (+18%; Zak et al., 2008). These biochemical responses have occurred in parallel with a change in the composition of saprotrophic fungi and bacteria inhabiting soil, as well as changes in the prominence of fungal and bacterial lignocellulose metabolism (Eisenlord and Zak, 2010; Edwards et al., 2011; Eisenlord et al., 2013; Freedman and Zak, 2014). Nonetheless, we do not understand how broader changes in the metabolic capacity of these soil organisms has been altered by anthropogenic N deposition, and furthermore, whether this response could foster greater soil C storage in our long-term field experiment.

A diverse assemblage of saprotrophic bacteria and fungi secrete extracellular enzymes that mediate the decay of cellulose, hemicellulose, lignin, and chitin, processes that largely control the cycling of C in terrestrial ecosystems (Osono, 2007; Voríšková et al., 2014). Understanding the molecular mechanisms by which anthropogenic N deposition modify the microbial metabolism of plant and fungal detritus is central to our ability to anticipate the storage and cycling of C in terrestrial ecosystems. For example, litter decay is largely mediated by lignocellulolytic enzymes of fungal origin and can result in complete metabolism to CO_2_ (Kirk and Farrell, 1987; D'souza et al., 1999; Polaina and Maccabe, 2007). Some bacteria also can participate in lignocellulose decay, which results in the production of soluble polyphenols (~60% of products) with minimal amounts of CO_2_ (< 4%; Godden et al., 1992; Ahmad et al., 2010; Bugg et al., 2011). Thus, how the composition and function of saprotrophic bacteria and fungi responds to anthropogenic N deposition has implications for the cycling and storage of C in the future. Shotgun metagenomics (i.e., the direct sequencing of environmental DNA) can be used to elucidate the genetic potential of complex microbial communities and provides the opportunity to simultaneously explore the taxonomic membership and functional capacity of the community (Fierer et al., 2012; Sharpton, 2014). This technology can provide insight into microbially mediated biogeochemical cycles, but it has rarely been applied to replicated experimental studies of soil microbial communities (Prosser, 2010; Knight et al., 2012; Myrold et al., 2014). Moreover, metagenomic studies in soil have mainly focused on bacteria, although recent improvements to genomic databases have allowed for metagenomic insights into the fungal community as well (Cline and Zak, 2015; Hesse et al., 2015).

Here, we employed shotgun metagenomics and extracellular enzyme assays in a well-replicated, long-term field experiment to decipher whether nearly two decades of experimental N deposition has altered the metabolic capacity of the soil bacterial and fungal community to metabolize plant and fungal detritus. It is plausible that chronic N deposition can modify the metabolic capacity of the microbial community that mediates decay, which may elicit a functional response consistent with our biogeochemical observations (Zak et al., 2008; Eisenlord et al., 2013). We used ribosomal and functional gene databases (i.e., RDP and Subsystems, respectively in MG-RAST) to gain insight into whether experimental N deposition altered the taxonomic composition, as well as the metabolic capacity of the soil microbial community. Sequence homology with manually curated gene databases was also employed to explore the genetic capacity of both bacteria and fungi to metabolize lignocellulose (Cline and Zak, 2015). Using these approaches, we obtained insight to the taxonomic composition and metabolic capacity of saprotrophic bacteria and fungi in response to anthropogenic N deposition in a long-term field study.

## Materials and methods

### Site description and sample collection

We investigated the influence of experimental N deposition on the functional capacity of the forest floor microbial community in four northern hardwood forest stands in Lower and Upper Michigan, USA (Table 1; Figure S1). The stands span the north-south geographic range of the northern hardwood forests in the Great Lakes region (Braun, 1950) and lie along a climatic and atmospheric N deposition gradient. All sites are dominated by sugar maple (Acer saccharum Marsh.) and are otherwise floristically and edaphically similar. The thin Oi horizon is mainly comprised of sugar maple leaf litter and the Oe/Oa horizon is interpenetrated by a dense mat of fine roots (~0.5 mm dia.); the Oe/Oa material was used in this study and it is where decay has been reduced. Soils are sandy (85–90%), well-drained, isotic, frigid Typic Haplorthods of the Kalkaska series. Six plots (30 × 30 m) were established at each stand in 1994; three receive ambient N deposition and three receive experimental N deposition. Experimental N deposition consists of NaNO_3_ pellets broadcast over the forest floor in six equal applications during the growing season (30 kg N ha^−1^year^−1^); in our study sites, ${\text{NO}}_{3}^{-}$-N comprises ~60% of atmospheric N deposition (Zak et al., 2008; Barnard et al., 2013).

**Table 1:** **Site, climatic, overstory, and ambient nitrogen deposition rates of four study sites receiving experimental N additions**.

**Characteristic**	**Site A**	**Site B**	**Site C**	**Site D**
**LOCATION**				
Latitude (N)	46°52”	45°33”	44°23”	43°40”
Longitude (W)	88°53”	84°52”	85°50”	86°9”
**CLIMATE**				
Mean annual temperature (°C)	4.8	6.1	6.5	7.7
Mean annual precipitation (cm)	91.9	93.3	92.8	86.6
Ambient N Deposition (Kg N ha^−1^ year^−1^)	5.9	6.1	7.4	7.4
**VEGETATION**				
Overstory biomass (Mg ha^−1^)	261	261	274	234
*Acer saccharum* biomass (Mg ha^−1^)	237	224	216	201
**ENVIRONMENT**				
**Leaf Litter (Oe/Oa horizons)**				
Litter C:N	63.7	57.1	52.9	43.4
Litter mass (g)	412	396	591	550
**Soil (0–10 cm)**				
Sand (%)	85	89	89	87
pH (1:1 soil/H_2_O)	4.8	5.0	4.5	4.7
Base saturation, %	71	96	73	80

*Data represent site, climatic, overstory, and ambient nitrogen deposition rates of four study sites receiving experimental N additions. Reprinted by Permission, ASA, CSSA, SSSA*.

Forest floor sampling occurred in late May to early June 2013. Samples from all four sites were taken during a phenologically similar period, a time at which ample moisture supports high rates of microbial activity. Within each 30 × 30-m plot, 10 random 0.1 × 0.1-m forest floor samples (Oa/Oe horizons) were collected after removing the Oi horizon. All samples were composited within each plot and hand homogenized in the field. A portion of the homogenized sample was immediately frozen in liquid N_2_ for nucleic acid extraction; the remainder was kept on ice for enzyme analyses. Samples were transported to the University of Michigan, where they were stored at −4 or −80°C for enzyme analysis and nucleic acid extraction, respectively.

### Enzyme analysis

We measured the potential activity of extracellular enzymes that catalyze the metabolism of two common plant litter compounds, cellulose (cellobiohydrolase; EC 3.2.1.91), and lignin (peroxidase, EC 1.11.1.7; phenol oxidase, EC 1.10.3.2). We also assayed β-1,4-N-acetylglucosaminidase (NAGase; EC 3.1.6.1), which catalyzes the metabolism of chitin, a component of fungal cell walls. Enzyme assays were initiated 72 h after field sampling and were performed according to Saiya-Cork et al. (2002). Briefly, a fluorometric assay was used to determine cellobiohydrolase and NAGase activity, in which methylumbelliferone-linked cellobioside or N-acetylglucosamine was used as the respective substrates. Peroxidase and phenol oxidase activity was measured using a colorimetric assay with L-dihydroxyphenylalanine (L-DOPA) as the substrate with the addition of hydrogen peroxide for the peroxidase assay. All enzyme assays were performed in 96-well plates on a Synergy HT microplate reader (Gen5, version 2.00.18, BioTek, Winooski, VT, USA). Enzyme activity potential was defined as units, wherein 1 unit = 1 μmol h^−1^ of the oxidized reaction product relative to the dry weight of forest floor. The effects of location, experimental N deposition, and their combined interaction on enzyme activity potential was determined by analysis of variance (ANOVA); means were compared with a protected Fisher's LSD in the R environment using the stats package (Version 3.01; R Code Team, 2013).

### DNA extraction and metagenome generation

Genomic DNA was extracted from 0.60 g (total fresh weight; six replicate extractions) of homogenized forest floor samples using a PowerLyzer PowerSoil DNA isolation kit with a PowerLyzer 24 homogenizer (MoBio Laboratories, Carlsbad, CA). To increase the quality and yield of nucleic acids, we modified the manufacturer's protocol by the initial addition of 250 μL phenol:chloroform:isoamyl alcohol (25:24:1; pH 6.7), bead beating at 4000 rpm for 45 s, centrifugation at 4°C; and overnight ethanol precipitation at −20°C. Extracted DNA was purified using a PowerClean DNA Cleanup kit (MoBio). Purified DNA quality was determined using an ND8000 Nanodrop (Thermo Scientific, Waltham, MA, USA) and quantified by Quant-iT PicoGreen (Invitrogen, Carlsbad, CA, USA) on a Synergy HT fluorometer.

Purified environmental DNA was submitted for shotgun sequencing at the University of Michigan DNA sequencing core. Samples were barcoded by plot (*n* = 24) and pooled in equi-molar concentrations on six lanes of an Illumina Hiseq 2500 (Illumina, San Diego, CA, USA), with 150 base single-end reads. Illumina reads containing adapter sequences were removed using Cutadapt version 1.7.1 (Martin, 2011). All metagenome sequence data have been deposited and are publically available in MG-RAST (Meyer et al., 2008) under accession numbers 4614815.3–4614838.3.

### Taxonomic identification

Taxonomic information was assessed from the shotgun metagenomes using the “best-hit classification” function within MG-RAST against the RDP database (Cole et al., 2014). Hits were taxonomically assigned using a maximum *e*-value of 1 × 10^−5^, minimum percent identity of 80%, and a minimum alignment length of 50 bp.

### Functional gene identification

To determine the impact of experimental N deposition on the metabolic potential of the forest floor microbial community, we considered the forest floor metagenomes as (i) functional pathways using a Subsystems-based approach in MG-RAST (Overbeek et al., 2014), (ii) bacterial lignocelluloytic gene composition using the SEED database, and (iii) fungal lignocelluloytic gene composition using BLASTN (Altschul et al., 1990) queries against manually-curated databases (Table S1; Cline and Zak, 2015). In this way, we obtained complimentary assessments of the lignocellulolytic capacity to determine how the forest floor microbial community may be affected by experimental N deposition. For Subsystems functional pathway analysis, metagenome hits were annotated using a maximum *e*-value of 1 × 10^−5^, a minimum percent identity of 60%, and a minimum alignment length cutoff of 25 amino acids. All metagenomes were first considered at broad classifications (i.e., Subsystem level one) to determine what functional categories may be affected by experimental N deposition. We further examined the level-one classifications “Carbohydrates,” “Metabolism of Aromatic Compounds,” and “Respiration” at a more resolved level (i.e., Subsystem level three) to gain further insight into a possible microbial mechanism reducing decay in our experiment. Throughout this manuscript, Subsystem level one categories will be termed “classifications” and Subsystem level 3 categories will be termed “pathways.” Gene assignments were standardized to the number of sequences with predicted functions (*sensu*; Fierer et al., 2012).

To determine the effect of experimental N deposition on specific functional genes mediating the decay of plant and fungal detritus, databases were constructed to determine the relative abundance of genes encoding enzymes mediating their metabolism. Metagenome sequences were assigned to 25 genes in 6 substrate categories (Table S1) using the SEED model within MG-RAST (Meyer et al., 2008). Due to the dominance of bacterial sequences in the MG-RAST databases, fungal metabolic potential was analyzed following the creation of 12 gene databases in 5 substrate categories, created collectively from the Carbohydrate Active Enzyme database (CAZy), Peroxibase, the Functional Gene Repository, and NCBI reference sequences (Fawal et al., 2013; Fish et al., 2013; Lombard et al., 2014; Tatusova et al., 2015). By doing so, the taxonomic assignment of DNA sequences enabled the creation of fungal gene databases separate and distinct from bacteria (*sensu*; Cline and Zak, 2015). Functional gene sequences were accessed from each database on January 13–15, 2015 and are available for download on GitHub (https://github.com/zacf/UM-gradient-metagenome-databases).

Sequences from shotgun metagenomes were assigned to functional genes by substrate category, including genes associated with the decay of cellulose, chitin, galactose-containing oligosaccharides, lignin, pectin, starch, and xylan (Table S1). For each metagenome, the abundance of genes involved in the decay of each substrate category (e.g., cellulose, lignin, xylan) was calculated following the assignment of metagenome sequences to functional gene databases using BLASTN. Bacterial and fungal genes were assigned using 60% minimum sequence homology, *e*-value cut-off of 1 × 10^−5^, minimum alignment 30 bp. Gene assignments were standardized to the number of sequences with predicted functions for each metagenome (*n* = 24).

### Statistical analyses of metagenomic data

Prior to statistical analysis, only those gene families that occurred in each of the 4 forest stands exposed to either ambient or experimental N deposition were chosen for further analysis to reduce the inclusion of erroneously annotated reads. For sequences where multiple Subsystem or gene categories were assigned, all categories were counted as additional hits (*sensu*; Žifcáková et al., 2016). All univariate analyses were performed in the R environment (Version 3.01; R Code Team, 2013). Functional and taxonomic metagenome abundance tables were normalized using the “normalize” function in matR prior to statistical analysis (Version 1.0.0; Braithwaite and Keegan, 2013). The change in relative abundance of hits attributed to functional genes and pathways were assessed by two-way ANOVA with site, treatment, and their interaction as factors. When applicable, *P*-values were corrected for multiple comparisons using the Benjamini and Hochberg False Discovery Rate correction (Benjamini and Hochberg, 1995). In this circumstance, an “adjusted P” value is presented.

Calculation of beta-diversity indices and associated statistics were executed in Primer (version 6, Primer-E Ltd., Plymouth, UK). A Euclidian distance matrix based on matR normalized metagenome abundance was generated, from which the significance of compositional differences between microbial functional assemblages exposed to ambient and experimental N deposition were determined by permutational multivariate analysis of variance (PerMANOVA; Anderson, 2001). Contributions of each functional pathway to the multivariate dissimilarity between metagenomes under ambient and experimental N deposition were determined using Similarity Percentage analysis (SIMPER; Clarke, 1993).

## Results and discussion

### A sustained decline in lignolytic potential under experimental N deposition

**Figure 1:**
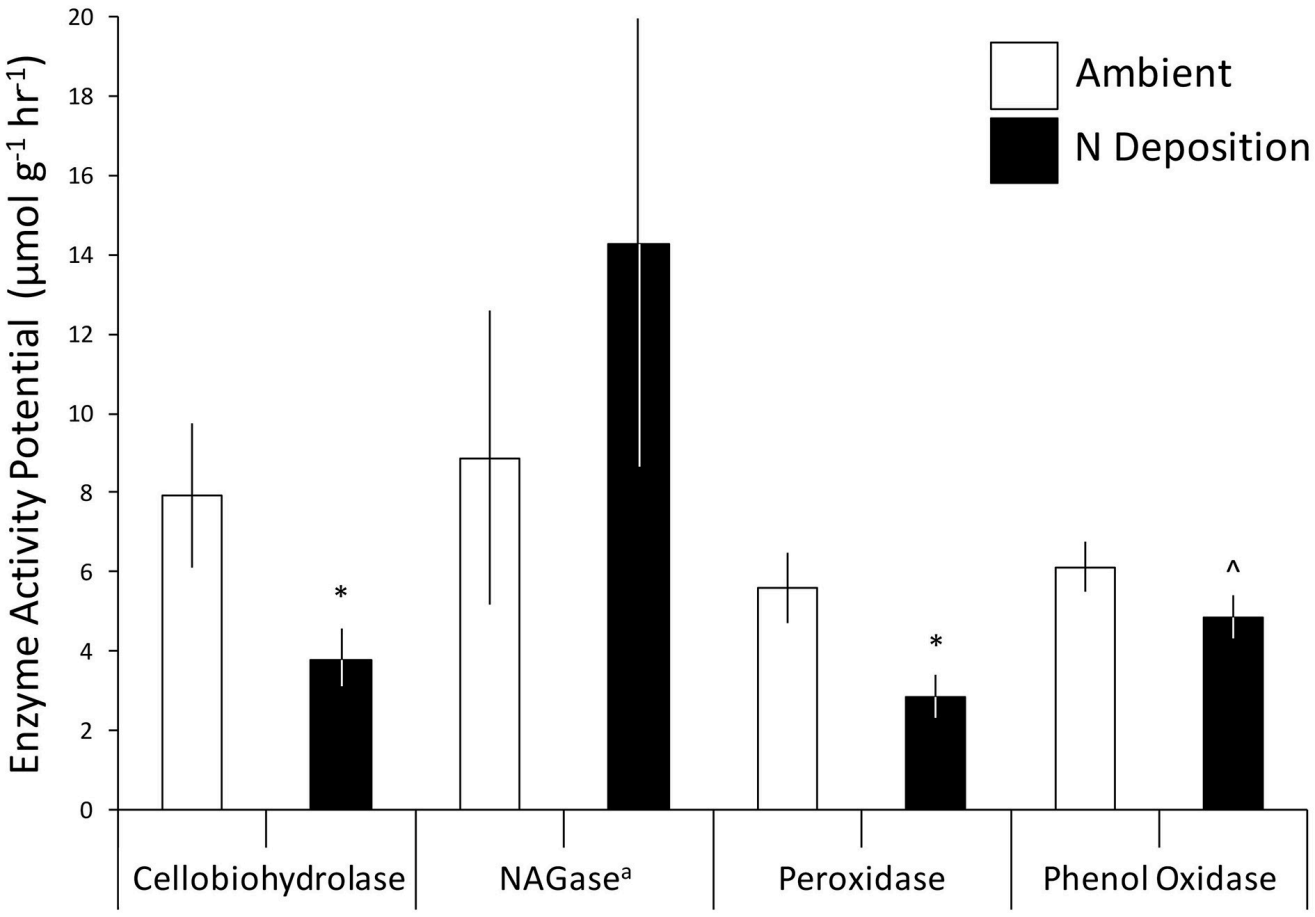
**The effect of experimental N deposition on microbial exo-enzyme activity potential in forest floor**. Values are expressed relative to the leaf litter dry weight. Mean ± standard error (*n* = 12) values are presented, eight analytical replicates were included. ^a^N-acetyl-glucosaminidase. ^*^P < 0.05 and ^∧^*P* < 0.10.

Experimental N deposition reduced lignocellulolytic enzyme activity and changed the metagenomic composition of the microbial community inhabiting decaying forest floor, providing indirect evidence that compositional shifts elicited a functional response in our long-term field experiment. The activity of two extracellular enzymes mediating plant litter and humus decay, cellobiohydrolase (−52% change from ambient; *P* = 0.02; Figure 1) and peroxidase (−50%; *P* = 0.01), were significantly lower under experimental N deposition. Although phenol oxidase exhibited the same response, the reduction of its activity under experimental N deposition was not statistically significant (−21%; *P* = 0.10). N-acetyl-glucosaminidase (NAGase) activity, which catalyzes the metabolism of chitin, trended higher under experimental N deposition, but this increase also was not significant (+61%; *P* = 0.37). Although, mean enzyme activities varied across the four sites (*P* < 0.05), experimental N deposition negatively affected cellobiohydrolase, peroxidase, and phenol oxidase activity similarly, as we observed no significant site by treatment interaction.

The results we present here are consistent with previous observations that experimental N deposition has reduced the activities of extracellular enzymes mediating plant litter and humus decay (Deforest et al., 2004, 2005; Waldrop et al., 2004; Edwards et al., 2011). In this study, cellobiohydrolase activity was lower under experimental N deposition as compared to the ambient treatment (Figure 1); whereas, it was previously determined that experimental N deposition leads to lower cellobiohydrolase activity, though non-significantly (Edwards et al., 2011). It is plausible that this sustained decline in enzymatic activity has decreased microbial metabolism of plant litter and humus, resulting in soil organic matter (SOM) accumulation and dissolved organic carbon (DOC) leaching previously documented in our long-term field experiment.

### Taxonomic assessment of the soil microbiome

We analyzed high-quality short reads to evaluate whether experimental N deposition has affected the taxonomic composition and functional capacity of the microbial communities inhabiting decaying forest floor. Sequencing and quality control produced 717,933,077 high-quality reads totaling 108.4 Gbases. Across the 24 metagenomes (e.g., 12 plots receiving ambient N deposition and 12 plots receiving experimental N deposition), 26–33% could be assigned to functional categories, percentages that are similar to other metagenomic surveys of soil samples (Fierer et al., 2012; Uroz et al., 2013).

We assessed the taxonomic classification of metagenomic hits using the RDP database within MG-RAST, and as expected, the metagenomes were dominated by Bacteria (~98% of annotated reads). Fungi represented 1.0 ± 0.6% of annotated reads, whereas Archaea represented 0.03 ± 0.007% of reads. The metagenomes were dominated by the bacterial phyla Actinobacteria (Figure S2; ~46%), Proteobacteria (~26%), and Bacteroidetes (~11%). However, the taxonomic composition of the soil microbial community was not significantly affected by experimental N deposition at the phylum (PerMANOVA; P = 0.27), class (P = 0.30), or species (P = 0.21) level. These observations provide evidence that the biogeochemical responses we have documented arise from a reduction in functional capacity of the extant community under experimental N deposition, rather than a shift in taxonomic composition that elicited a concomitant reduction in function. It also is consistent with a recently completed study demonstrating the experimental N deposition did not alter the total community of soil bacteria or fungi, but did modify the community of organisms who were active under experimental N deposition (Freedman et al., 2015).

The dominance of Bacteria in annotated reads from soil metagenomes is common (~83-98%; Fierer et al., 2012; Uroz et al., 2013; Navarrete et al., 2015), because annotation databases are biased toward culturable bacteria. Moreover, it is plausible that the underrepresentation of fungal genes in annotation databases leads to low coverage of fungi in soil metagenomic analyses (Wooley et al., 2010; Myrold et al., 2014). In addition to annotation biases, many fungal genes contain intronic sequences, which require long reads for accurate annotation. For this reason, the fungal contribution to soil metabolic function may be further resolved using a transcriptomic approach, as evidenced by a higher proportion of fungal transcripts in recent soil metatranscriptomes (~73%; Hesse et al., 2015; Žifcáková et al., 2016) as compared to the proportion of fungal genes in soil metagenomes (2-17%).

### Effects of N deposition on functional pathways of the soil microbiome

All metagenomes were initially assessed from a broad to fine level of functional resolution using Subsystems classifications within MG-RAST. At the broadest category (i.e., level 1), there was no difference in the relative abundance (Adjusted P > 0.10) or composition (Figure S3; PerMANOVA; P = 0.60) of hits assigned to each Subsystem (n = 28 Subsystem level 1 classifications). To further understand the impact of chronic N deposition on the genes mediating organic matter decay, we chose to focus our analysis on metagenomic hits assigned to the following Subsystem level one classifications: Carbohydrates, Metabolism of Aromatic Compounds, and Respiration. Within these Subsystem-level-one classifications, we analyzed the change in relative abundance of genes associated with each level-three pathway.

**Figure 2:**
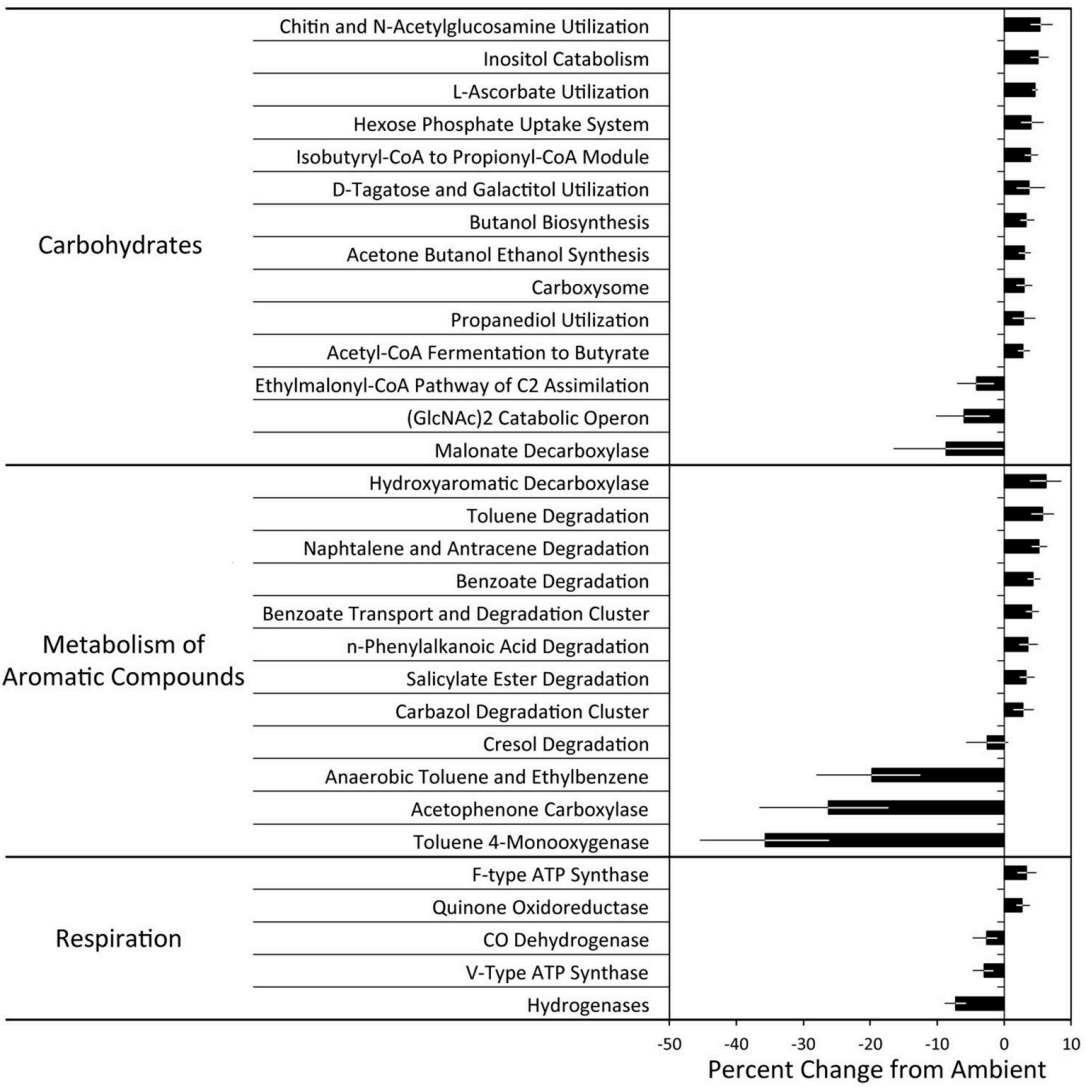
**The effect of experimental N deposition on the relative abundance of SEED subsystem level 3 functional pathways**. All pathways presented were significantly different in abundance and exhibited a greater than ±2.5% change between the ambient and experimental N deposition treatment (adjusted *P* < 0.05). Results of all significant pathways can be found in Tables S2, S3, and S4.

**Figure 3:**
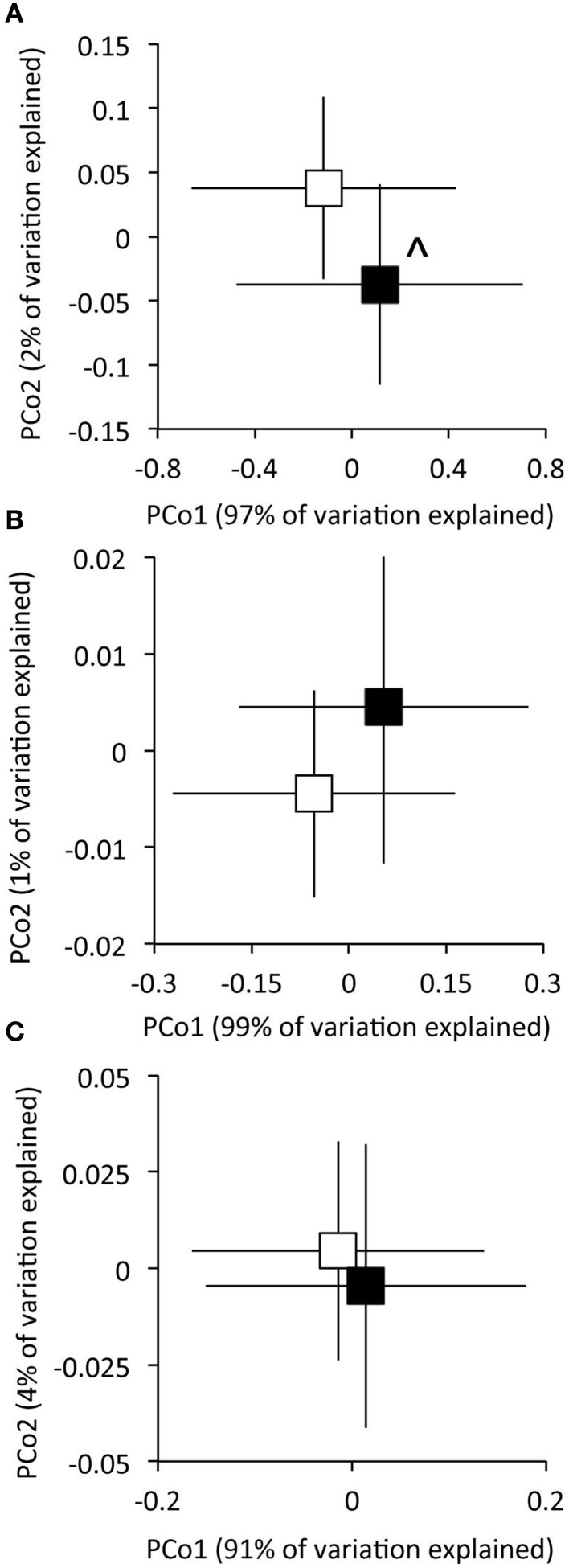
**The effect of experimental N deposition on the composition of functional gene pathways within the Carbohydrates (A), Metabolism of Aromatic Compounds (B), and Respiration (C) SEED subsystem classifications**. Ordinations were obtained from Principle Coordinates Analysis on based Euclidian distances of matR normalized data. Open and closed boxes indicate the composition of functional gene pathways in the ambient and experimental N deposition treatment, respectively. ^∧^*P* = 0.057 by PerMANOVA.

Experimental N deposition significantly and differentially affected the relative abundances of genes associated with 32 of 103 total pathways within the Carbohydrate classification (adjusted P < 0.05; Figure 2, Table S2). The composition of functional pathways within the Carbohydrates classification was also affected by experimental N deposition (PerMANOVA; P = 0.057; Figure 3A). To determine the relative contribution of gene pathways within the Carbohydrate classification to compositional dissimilarity between ambient and experimental N deposition assemblages, SIMPER analysis was performed (Table 2). SIMPER determined that pathways mediating unknown carbohydrate utilization (26.8% of community dissimilarity), malonate decarboylation (12.6%), and GlcNAc_2_ catabolism (10.1%) contributed the greatest to dissimilarity in the functional capacity of the microbial community exposed to ambient and experimental N deposition. All sites were affected by experimental N deposition in the same fashion, as there were no significant site by treatment interactions.

**Table 2:** **Contribution of gene pathways to compositional dissimilarity of the Carbohydrates classification as indicated by SIMPER analysis**.

**Variable**	**Contrib%**	**Cum.%**
Unknown carbohydrate utilization^a^	26.8	26.8
Malonate decarboxylase	12.6	39.4
GlcNAc_2_ Catabolic Operon	10.1	49.5
Ethylmalonyl-CoA pathway of C2 assimilation	8.3	57.7
Hexose Phosphate Uptake System	8.1	65.9
L-ascorbate utilization	7.6	73.5
L-fucose utilization	5.6	79.1
Xyloglucan utilization	3.9	83.0
Alpha-Amylase locus in Streptocococcus	2.0	85.0
Soluble methane monooxygenase (sMMO)	1.6	86.5

a *containing Fructose-bisphosphate aldolase*.

The forest floor metagenomes indicated that gene pathways mediating bacterial carbohydrate metabolism were favored under experimental N deposition. For example, of the significant Subsystem level 3 pathways, 27 of 32 increased in abundance in the experimental N deposition treatment. Among the most positively affected by experimental N deposition were genes mediating chitin utilization (~5% change from ambient; adjusted P = 0.05); this change occurred concomitantly with a non-significant increase in NAGase activity (+61%; P = 0.37; Figure 1). Chitin is a primary component of fungal cell wall; thus, an increase in genes meditating its metabolism may suggest greater fungal necromass under future rates of N deposition. Understanding how N deposition affects the decay of microbial necromass can help predict how this important, although poorly understood, C pool in soil may be altered by climate change (Clemmensen et al., 2013; Crowther et al., 2015). Genes mediating L-ascorbate utilization also increased in abundance under N deposition (~5%; adjusted P = 0.01) and accounted for ~8% of the functional dissimilarity between the microbial communities exposed to ambient and experimental N deposition. L-ascorbate (i.e., vitamin C) is an important metabolite for most living organisms. Thus, given the dominance of bacterial reads in the MG-RAST annotated metagenomes (i.e., ~98%), increased utilization of L-ascorbate under N deposition supports the hypothesis that bacterial metabolism could be favored under future rates of N deposition, and may mediate increased C storage in our long-term field experiment.

While many of the significantly affected gene pathways mediating carbohydrate metabolism increased in abundance in response to experimental N deposition, some responded negatively. For example, experimental N deposition reduced the abundance of genes mediating malonate decarboxylation (−9%; adjusted P = 0.04), which also accounted for ~13% of the functional dissimilarity between the microbial communities exposed to ambient and experimental N deposition. Malonate is a common organic acid in soil; it is a TCA cycle intermediate and also is involved in soil carbon dynamics and nutrient solubilization (Oburger et al., 2011). We also observed a 6% decrease in the catabolic operon of the chitin disaccharide, N,N-diacetylchitobiose (GlcNAc_2_; adjusted *P* = 0.01), which accounted for ~10% of the dissimilarity between the functional capacity of the microbial communities exposed to ambient and experimental N deposition. Bacterial chitin degradation pathways are diverse and phylogenetically widespread (Beier and Bertilsson, 2013; Zimmerman et al., 2013). While genes encoding chitinases are well-represented in functional gene databases, much of what is known of gene-operons conferring GlcNAc_2_ degradation capability (e.g., regulators and transporters) is from organisms inhabiting the marine environment (e.g., *Vibrio sp*.; Meibom et al., 2004). Thus, this result is likely indicative of an ecologically irrelevant subset of operonic genes and not of the entire community.

Within the Metabolism of Aromatic Compounds (MAC) Subsystem level one classification, experimental N deposition significantly affected genes associated with 21 of 32 level three functional pathways (Figure 2, Table S3). All sites were affected by experimental N deposition in the same fashion, as there were no significant site by treatment interactions. The composition of functional pathways within the Metabolism of Aromatic Compounds classification was not affected by experimental N deposition (Figure 3B; PerMANOVA; P = 0.26).

Similar to carbohydrate metabolism, particular pathways of bacterial aromatic compound metabolism were favored under future rates of N deposition. Of the significant level three pathways, 16 of 21 increased in abundance under experimental N deposition. Among the most positively affected were the hydroxyaromatic decarboxylase and benzoate degradation pathways, which increased in abundance under experimental N deposition by 6 and 4% respectively, as compared to the ambient treatment (adjusted P < 0.05). This suggests an increased importance of bacterial decarboxylation of hydroaromatic compounds (e.g., benzoate) under future rates of N deposition. Genes mediating anaerobic toleuene degradation and toluene-4-monooxygenase were most negatively affected by experimental N deposition (−20 and −36% change from ambient, respectively; adjusted P < 0.05), but likely are ecologically irrelevant differences in the microbial functional capacity due to the well-drained nature of the sandy soils in our experiment. We did not expect much, if any toluene, to be present in the soils of our study, and the emission of toluene during litter decomposition is negligible (Gray et al., 2010). Given the similarity of many of these compounds with poly-phenolic compounds whose leaching has increased under experimental N deposition (Pregitzer et al., 2004), the increased metabolism of aromatic compounds will be a favored microbial trait as rates of N deposition continue to increase in the future (Freedman and Zak, 2014).

The relative abundance of genes associated with 15 of 42 level three pathways within the Respiration classification were significantly affected by experimental N deposition (Figure 2; Table S4). All sites were affected by experimental N deposition in the same fashion, as there were no significant site by treatment interactions. The composition of functional pathways within the Respiration classification also was not affected by experimental N deposition (Figure 3C; PerMANOVA; P = 0.42).

Analysis of functional pathways within the Respiration classification was mixed, as 7 of 15 significant level three pathways increased in abundance in the experimental N deposition treatment (Figure 2). For example, the abundance of genes encoding bacterial and eukaryotic ATPases, enzymes that dephosphorylate ATP, were differentially affected by experimental N deposition. Experimental N deposition led to an increase in the relative abundance of bacterial (F-type; +3%; adjusted P = 0.04) and a decrease in eukaryotic ATPases (V-type; −3%; adjusted P = 0.01). These results support a stimulation of saprotrophic bacterial activity and a suppression of fungi under future rates of N deposition (Edwards et al., 2011; Freedman and Zak, 2014, 2015b). Genes encoding hydrogenases were most negatively affected (−7%; adjusted P = 0.03) by the experimental N deposition treatment. N_2_-fixing soil microorganisms release substantial amounts of H_2_ as a byproduct of nitrogenase activity, which is suppressed by N fertilization. (Dasilva et al., 1993; Freedman et al., 2013; Berthrong et al., 2014). It is possible that a suppression of microbial N_2_ fixation is occurring with experimental N deposition, leading to less H_2_ emission in soils and lower hydrogenase activity. While soil represents the largest sink of atmospheric H_2_, contributing about 75% to the total budget (Prather, 2003), we do not expect the biochemical pathway to have any effect on soil C storage in our long-term field experiment.

### Effect of simulated N deposition on the abundance of functional genes mediating SOM decay

We estimated the relative abundance of individual bacterial and fungal lignocellulolytic genes mediating litter decay using a curated database of genes (Table S1). This enabled us to assess how experimental N deposition affects specific genes encoding enzymes mediating lignocellulose decay. Experimental N deposition significantly reduced the relative abundance of bacterial genes mediating the decay of chitin (Figure S4; −8.5%; adjusted P = 0.03), and this effect was consistent across sites (site × treatment; adjusted P = 0.11). The relative abundance of bacterial genes mediating the decay of other substrates (i.e., cellulose, hemicellulose, lignin, pectin, and starch) was not affected by experimental N deposition. When considered together, the composition of bacterial lignocellulolytic functional genes was affected in a site-specific manner, as indicated by a significant site by treatment interaction (Figure S5; PerMANOVA; P = 0.03). Pairwise tests indicate that experimental N deposition altered the composition of bacterial lignocellulolytic genes in Sites B (Pairwise P = 0.01) and D (Pairwise P = 0.01), but not in Sites A and C (Pairwise P > 0.20). This contrasts with previous work in which we observed and increased relative abundance of bacterial LMCO genes under experimental N deposition (Freedman and Zak, 2014). Bacterial communities exhibit seasonal community dynamics, as well as successional changes during leaf decay (Lipson and Schmidt, 2004; Torres et al., 2005; Chapman et al., 2013; Freedman and Zak, 2015a). It is plausible that saprotrophic bacteria respond to experimental N deposition, as well as other agents of environmental change, in a seasonally or temporally dependent manner. To more definitively understand the bacterial response to future rates of N deposition, we must gain a greater understanding of the temporal robustness of their response.

The relative abundance of fungal genes mediating cellulose, hemicellulose, lignin, pectin, and xylan decay also were not affected by experimental N deposition (adjusted P > 0.05). When considered together, the composition of fungal functional genes was not affected by experimental N deposition (PerMANOVA; P = 0.42); however, the composition of fungal lignocellulolytic genes did differ between sites (P = 0.02).

Experimental N deposition has altered the bacterial lignocellulolytic potential in a site-dependent manner; whereas, the functional potential of the saprophytic fungal community is unaffected by chronic N deposition. However, previous observations indicate that soil fungi down-regulate the expression of gene with lignolytic function under experimental N deposition (Edwards et al., 2011; Hesse et al., 2015). Results presented here supports the hypothesis that the lignocellulolytic potential of the fungal community was not affected by future rates of N deposition; however, the expression of these fungal genes (i.e., the active community) was negatively effected by this agent of environmental change (Edwards et al., 2011; Freedman et al., 2015).

### Toward a microbial mechanism mediating increased C storage under future rates of N deposition

Results presented here indicate that future rates of N deposition alter the functional capability of the soil microbial community inhabiting decaying leaf litter, namely, the suppression of the lignocellulolytic enzymatic capacity of the soil microbial community, and the increased prevalence of bacterial gene pathways implicated in litter decay. These enzymatic and metagenomic changes underlie a biogeochemical response, as nearly 20 years of experimental N deposition has reduced litter decay in our long-term experiment (Zak et al., 2008), indicative of a phenomenon commonly observed among terrestrial ecosystems exposed to simulated N deposition (Frey et al., 2004; Liu and Greaver, 2010; Maaroufi et al., 2015). The decreased enzyme activities we observed are consistent with the suppression of fungal laccase expression in our experiment (−80%; Edwards et al., 2011). Lignocellulolytic enzymes of fungal origin have a higher redox potential than their bacterial counterparts (Kirk and Farrell, 1987; D'souza et al., 1999; Polaina and Maccabe, 2007); thus, an increase in bacterial gene pathways mediating aromatic compound and carbohydrate metabolism may not be sufficient to offset the oxidative potential lost by a reduction in fungal lignocelluloytic gene expression. Further, future rates of N deposition favor gene pathways associated with saprotrophic bacteria, whose litter decay capacity is characterized by the production of soluble polyphenols with minimal CO_2_ production (Ahmad et al., 2010; Bugg et al., 2011). Data presented here together with previous work supports the hypothesis that the ascendance of saptrotrophic bacteria and the suppression of saprotrophic fungi may mediate increased C storage in our long-term field experiment (Edwards et al., 2011; Freedman and Zak, 2014).

## Conclusions

In this study, we present evidence that nearly 20 years of experimental N deposition has resulted in a sustained decline in the decay capability of the forest floor microbial community, which occurred concomitantly with changes in the relative abundance of functional pathways mediating the metabolism of carbohydrates and aromatic compounds, as well as respiration. Furthermore, bacterial gene pathways mediating the metabolism of carbohydrates and aromatic compounds were favored by experimental N deposition; whereas, the abundance and composition of fungal lignocellulolytic genes were unchanged by this agent of environmental change. Results presented here provide evidence that changes in the functional capacity of saprotrophic soil microorganisms mediate how anthropogenic N deposition slows the cycling and increases the storage of C in soil.

## Author contributions

DZ conceived the study. All authors carried out the research. ZF analyzed the data and prepared the first draft of the manuscript. All authors were involved in the revision of the draft manuscript and have agreed to the final content.

## Funding

Grants from the U. S. Department of Energy's BER program, the NSF LTREB program, and USDA McIntire-Stennis Program provided support for our work.

### Conflict of interest statement

The authors declare that the research was conducted in the absence of any commercial or financial relationships that could be construed as a potential conflict of interest.
