# Supplementary material for: Anthropogenic N Deposition Slows Decay by Favoring Bacterial Metabolism: Insights from Metagenomic Analyses
Source: Front Microbiol. 2016 Mar 2;7:259. doi: 10.3389/fmicb.2016.00259 (PMC4773658; doi:10.3389/fmicb.2016.00259)
Supplement: Supplementary file 1 [file Table1.DOCX]

**Supplementary Table S1.** Summary of functional genes used in metagenomic analysis of bacterial functional potential to degrade plant and microbial litter.

| **Community** | **Enzyme (Gene) Name** | **EC Number** | **Substrate Category** |
| --- | --- | --- | --- |
| Bacteria | Beta-glucosidase | 3.2.1.21 | Cellulose |
|  | Endocellulase | 3.2.1.4 | Cellulose |
|  | Alpha-N-acetylglucosaminidase | 3.2.1.50 | Chitin |
|  | Chitin deacetylase | 3.5.1.41 | Chitin |
|  | Endochitinase | 3.2.1.14 | Chitin |
|  | β-N-acetylhexosaminidase | 3.2.1.52 | Chitin |
|  | acetyl xylan esterase, putative, axe2B | 3.1.1.72 | Hemicellulose |
|  | Alpha galactosidase | 3.2.1.22 | Hemicellulose |
|  | Alpha-glucuronidase | 3.2.1.139 | Hemicellulose |
|  | Alpha-L-arabinofuranosidase | 3.2.1.55 | Hemicellulose |
|  | Beta-galactosidase | 3.2.1.23 | Hemicellulose |
|  | Beta-mannosidase | 3.2.1.25 | Hemicellulose |
|  | Beta-xylosidase | 3.2.1.37 | Hemicellulose |
|  | Endo-1,4-ß-xylanases | 3.2.1.8 | Hemicellulose |
|  | feruloyl esterase | 3.1.1.73 | Hemicellulose |
|  | Mannan endo-1,4-beta-mannosidase B precursor | 3.2.1.78 | Hemicellulose |
|  | Laccase | 1.10.3.2 | Lignin |
|  | Endopolygalacturonase | 3.2.1.15 | Pectin |
|  | Exopolygalacturonase / galacturan 1,4-α-galacturonidase | 3.2.1.67 | Pectin |
|  | Pectate lyase | 4.2.2.2 | Pectin |
|  | Pectin lyase | 4.2.2.10 | Pectin |
|  | Pectinesterase | 3.1.1.11 | Pectin |
|  | Alpha-amylase | 3.2.1.1 | Starch |
|  | Alpha-glucosidase | 3.2.1.20 | Starch |
|  | Glucan 1,4-alpha-glucosidase | 3.2.1.3 | Starch |
| Fungi | B-glucosidase | 3.2.1.21 | Cellulose |
|  | Cellulose 1,4-beta-cellobiosidase (cbh1) | 3.2.1.91 | Cellulose |
|  | α-N-acetylglucosaminidase | 3.2.1.50 | Chitin |
|  | β-N-acetylhexosaminidase | 3.2.1.52 | Chitin |
|  | Alpha galactosidase | 3.2.1.22 | Hemicellulose |
|  | Alpha-L-arabinofuranosidase | 3.2.1.55 | Hemicellulose |
|  | Beta-xylosidase | 3.2.1.37 | Hemicellulose |
| **Supplementary Table S1, continued** | | | |
| **Community** | **Enzyme (Gene) Name** | **EC Number** | **Substrate Category** |
|  | Laccase (lcc) | 1.10.3.2 | Lignin |
|  | Lignin peroxidase (lip)* | 1.11.1.14 | Lignin |
|  | Manganese peroxidase (mnp)* | 1.11.1.13 | Lignin |
|  | Versatile peroxidase (vp)* | 1.11.1.16 | Lignin |
|  | Endopolygalacturonase | 3.2.1.15 | Pectin |

* Due to similarities in functional genes and relatively short reads from Illumina sequencing, a single gene database was created.
