# Supplementary material for: Anthropogenic N Deposition Slows Decay by Favoring Bacterial Metabolism: Insights from Metagenomic Analyses
Source: Front Microbiol. 2016 Mar 2;7:259. doi: 10.3389/fmicb.2016.00259 (PMC4773658; doi:10.3389/fmicb.2016.00259)
Supplement: Supplementary file 2 [file Table2.DOCX]

**Supplementary Table S2.** Change in the relative abundance of Carbohydrates Subsystem level 3 pathways due to experimental N deposition.

| Level 3 | % Change from Ambient | Corrected P-Value |
| --- | --- | --- |
| GlcNAc_2_ Catabolic Operon | -6.0 ± 4.3 | 0.01 |
| Acetone Butanol Ethanol Synthesis | 3.0 ± 1.1 | 0.01 |
| Acetyl-CoA Fermentation to Butyrate | 2.8 ± 1.2 | 0.01 |
| Alpha-Amylase Locus in Streptocococcus | 1.5 ± 1.7 | 0.01 |
| Beta-Glucoside Metabolism | 0.8 ± 0.9 | 0.00 |
| Butanol Biosynthesis | 3.2 ± 1.3 | 0.01 |
| Carboxysome | 2.9 ± 1.4 | 0.00 |
| Chitin and N-acetylglucosamine Utilization | 5.3 ± 1.9 | 0.05 |
| D-galactarate, D-glucarate and D-glycerate Catabolism | 1.8 ± 1.8 | 0.05 |
| D-Galacturonate and D-Glucuronate Utilization | 1.8 ± 1.3 | 0.02 |
| D-Gluconate and ketogluconates metabolism | -0.4 ± 1.7 | 0.03 |
| D-Tagatose and Galactitol Utilization | 3.7 ± 2.3 | 0.02 |
| Ethylmalonyl-CoA pathway of C2 assimilation | -4.1 ± 2.9 | 0.02 |
| Glyoxylate bypass | 0.2 ± 0.9 | 0.02 |
| Hexose Phosphate Uptake System | 4.0 ± 1.9 | 0.01 |
| HPr kinase and hprK operon | 1.6 ± 1.0 | 0.05 |
| Inositol catabolism | 5.0 ± 1.7 | 0.02 |
| Isobutyryl-CoA to Propionyl-CoA Module | 3.9 ± 1.3 | 0.01 |
| L-Arabinose utilization | 2.4 ± 0.9 | 0.00 |
| L-ascorbate utilization (and related gene clusters) | 4.5 ± 0.5 | 0.01 |
| L-fucose utilization | 1.0 ± 1.0 | 0.01 |
| L-fucose utilization temp | 1.0 ± 0.9 | 0.02 |
| Malonate decarboxylase | -8.7 ± 7.9 | 0.04 |
| Methylglyoxal Metabolism | 1.9 ± 1.0 | 0.01 |
| One-carbon metabolism by tetrahydropterines | 0.6 ± 0.6 | 0.01 |
| Propanediol utilization | 2.9 ± 1.8 | 0.02 |
| Propionate-CoA to Succinate Module | 2.5 ± 0.9 | 0.03 |
| Soluble methane monooxygenase (sMMO) | 2.0 ± 1.8 | 0.03 |
| Sucrose utilization | 0.1 ± 1.3 | 0.05 |
| Unknown carbohydrate utilization | -1.4 ± 0.7 | 0.02 |
| Xyloglucan Utilization | 0.7 ± 0.7 | 0.02 |
| Xylose utilization | 0.4 ± 1.0 | 0.01 |

Data represent the average ± SE of the percent change in relative abundance of each Subsystems level 3 functional pathway across the four experimental forest stands.
