# Supplementary material for: Anthropogenic N Deposition Slows Decay by Favoring Bacterial Metabolism: Insights from Metagenomic Analyses
Source: Front Microbiol. 2016 Mar 2;7:259. doi: 10.3389/fmicb.2016.00259 (PMC4773658; doi:10.3389/fmicb.2016.00259)
Supplement: Supplementary file 3 [file Table3.DOCX]

**Supplementary Table S3.** Change in the relative abundance of Metabolism of Aromatic Compounds Subsystem level 3 pathways due to experimental N deposition.

| Level 3 | % Change from Ambient | Corrected P-Value |
| --- | --- | --- |
| 4-Hydroxyphenylacetic acid catabolic pathway | 2.3 ± 1.20 | 0.00 |
| Acetophenone carboxylase 1 | -26.2 ± 10.3 | 0.01 |
| Anaerobic benzoate metabolism | 2.3 ± 1.00 | 0.00 |
| Anaerobic toluene degradation | -19.8 ± 8.30 | 0.01 |
| Benzoate degradation | 4.3 ± 1.10 | 0.00 |
| Benzoate transport and degradation cluster | 4.1 ± 1.10 | 0.01 |
| Biphenyl degradation | 0.3 ± 1.80 | 0.01 |
| Carbazol degradation cluster | 2.8 ± 1.60 | 0.04 |
| Central meta-cleavage pathway | 2.4 ± 1.50 | 0.01 |
| Chlorobenzoate degradation | 0.9 ± 0.80 | 0.00 |
| Cresol degradation | -2.6 ± 3.10 | 0.04 |
| Homogentisate pathway | 1.7 ± 1.40 | 0.01 |
| Hydroxyaromatic decarboxylase family | 6.2 ± 2.30 | 0.03 |
| n-Phenylalkanoic acid degradation | 3.5 ± 1.40 | 0.00 |
| Naphtalene and antracene degradation | 5.2 ± 1.20 | 0.01 |
| p-Hydroxybenzoate degradation | 0.9 ± 1.00 | 0.04 |
| Phenylacetyl-CoA catabolic pathway | -0.2 ± 2.30 | 0.01 |
| Quinate degradation | 1.2 ± 0.30 | 0.01 |
| Salicylate ester degradation | 3.2 ± 1.30 | 0.02 |
| Toluene 4-monooxygenase | -35.7 ± 9.70 | 0.01 |
| Toluene degradation | 5.7 ± 1.70 | 0.05 |

Data represent the average ± SE of the percent change in relative abundance of each Subsystems level 3 functional pathway across the four experimental forest stands.
