# Supplementary material for: Anthropogenic N Deposition Slows Decay by Favoring Bacterial Metabolism: Insights from Metagenomic Analyses
Source: Front Microbiol. 2016 Mar 2;7:259. doi: 10.3389/fmicb.2016.00259 (PMC4773658; doi:10.3389/fmicb.2016.00259)
Supplement: Supplementary file 4 [file Table4.DOCX]

**Supplementary Table S4.** Change in the relative abundance of Respiration Subsystem level 3 pathways due to experimental N deposition.

| Level 3 | % Change from Ambient | Corrected P-Value |
| --- | --- | --- |
| Anaerobic respiratory reductases | 1.6 ± 1.2 | 0.00 |
| Biogenesis of cytochrome c oxidases | 1.7 ± 1.4 | 0.05 |
| Carbon monoxide dehydrogenase maturation factors | -0.3 ± 2.3 | 0.01 |
| Carbon monoxide induced hydrogenase | -0.1 ± 3.4 | 0.00 |
| CO Dehydrogenase | -2.6 ± 2.1 | 0.01 |
| F0F1-type ATP synthase | 3.3 ± 1.4 | 0.02 |
| Hydrogenases | -7.2 ± 1.7 | 0.02 |
| Na^+^-translocating NADH-quinone oxidoreductase  and *rnf*-like group of electron transport complexes | -1.4 ± 2.7 | 0.04 |
| Quinone oxidoreductase family | 2.6 ± 1.2 | 0.00 |
| Soluble cytochromes and related electron carriers | 0.7 ± 1.0 | 0.00 |
| Succinate dehydrogenase | 1.7 ± 1.1 | 0.03 |
| Terminal cytochrome O ubiquinol oxidase | -0.5 ± 1.1 | 0.03 |
| Terminal cytochrome oxidases | -0.2 ± 1.0 | 0.01 |
| Ubiquinone Menaquinone-cytochrome c reductase complexes | 1.3 ± 1.1 | 0.00 |
| V-Type ATP synthase | -3.0 ± 1.7 | 0.01 |

Data represent the average ± SE of the percent change in relative abundance of each Subsystems level 3 functional pathway across the four experimental forest stands.
