# Supplementary material for: Anthropogenic N Deposition Slows Decay by Favoring Bacterial Metabolism: Insights from Metagenomic Analyses
Source: Front Microbiol. 2016 Mar 2;7:259. doi: 10.3389/fmicb.2016.00259 (PMC4773658; doi:10.3389/fmicb.2016.00259)
Supplement: Supplementary file 5 [file Image1.PDF]

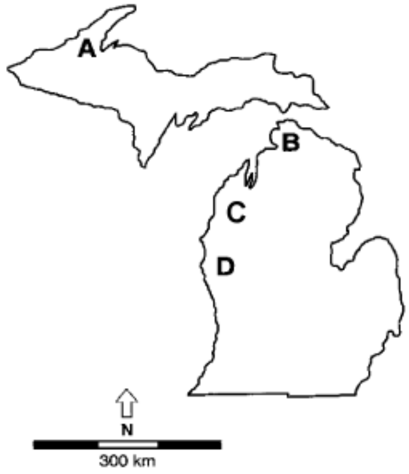

Supplementary Figure S1. The geographic distribution of the study sites in Lower and Upper Michigan. In each stand beginning in 1994, three plots received ambient atmospheric N deposition and three plots received ambient plus 30 kg NO_3^{-}–N ha^{-1} yr^{-1}.
