# Supplementary material for: Anthropogenic N Deposition Slows Decay by Favoring Bacterial Metabolism: Insights from Metagenomic Analyses
Source: Front Microbiol. 2016 Mar 2;7:259. doi: 10.3389/fmicb.2016.00259 (PMC4773658; doi:10.3389/fmicb.2016.00259)
Supplement: Supplementary file 6 [file Image2.PDF]

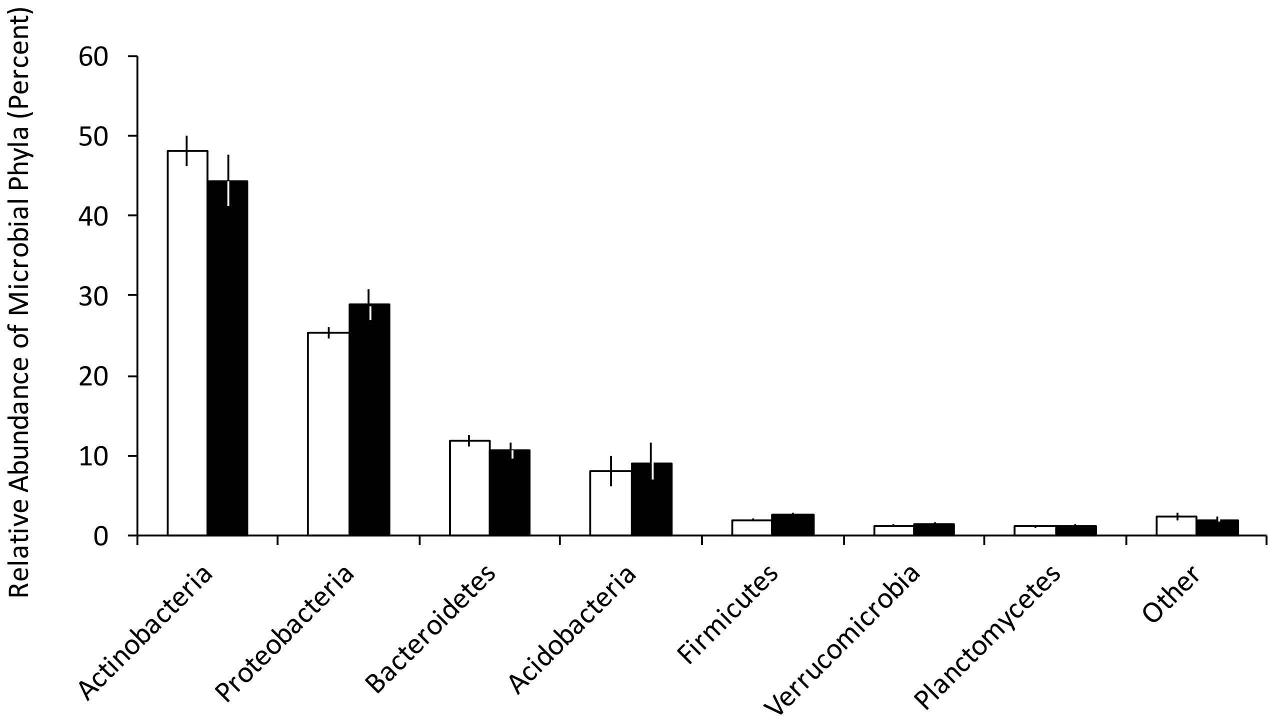

Supplementary Figure S2. The relative abundance of microbial phyla under ambient (white bars) and experimental N deposition (black bars) conditions. Data presented represent the mean ± SE (n=12). "Other" includes phyla with less than 1% relative abundance, including the bacterial phyla Chlamydiae, Cyanobacteria, Chloroflexi, Spirochaetes, Tenericutes, Gemmatimonadetes, Chlorobi, Thermotogae, Deferribacteres, and Deinococcus-Thermus, and Eukaryotes Ascomycota and Basidiomycota, Bacillariophyta and Archaea Thaumarchaeota.
