# Supplementary material for: Anthropogenic N Deposition Slows Decay by Favoring Bacterial Metabolism: Insights from Metagenomic Analyses
Source: Front Microbiol. 2016 Mar 2;7:259. doi: 10.3389/fmicb.2016.00259 (PMC4773658; doi:10.3389/fmicb.2016.00259)
Supplement: Supplementary file 7 [file Image3.PDF]

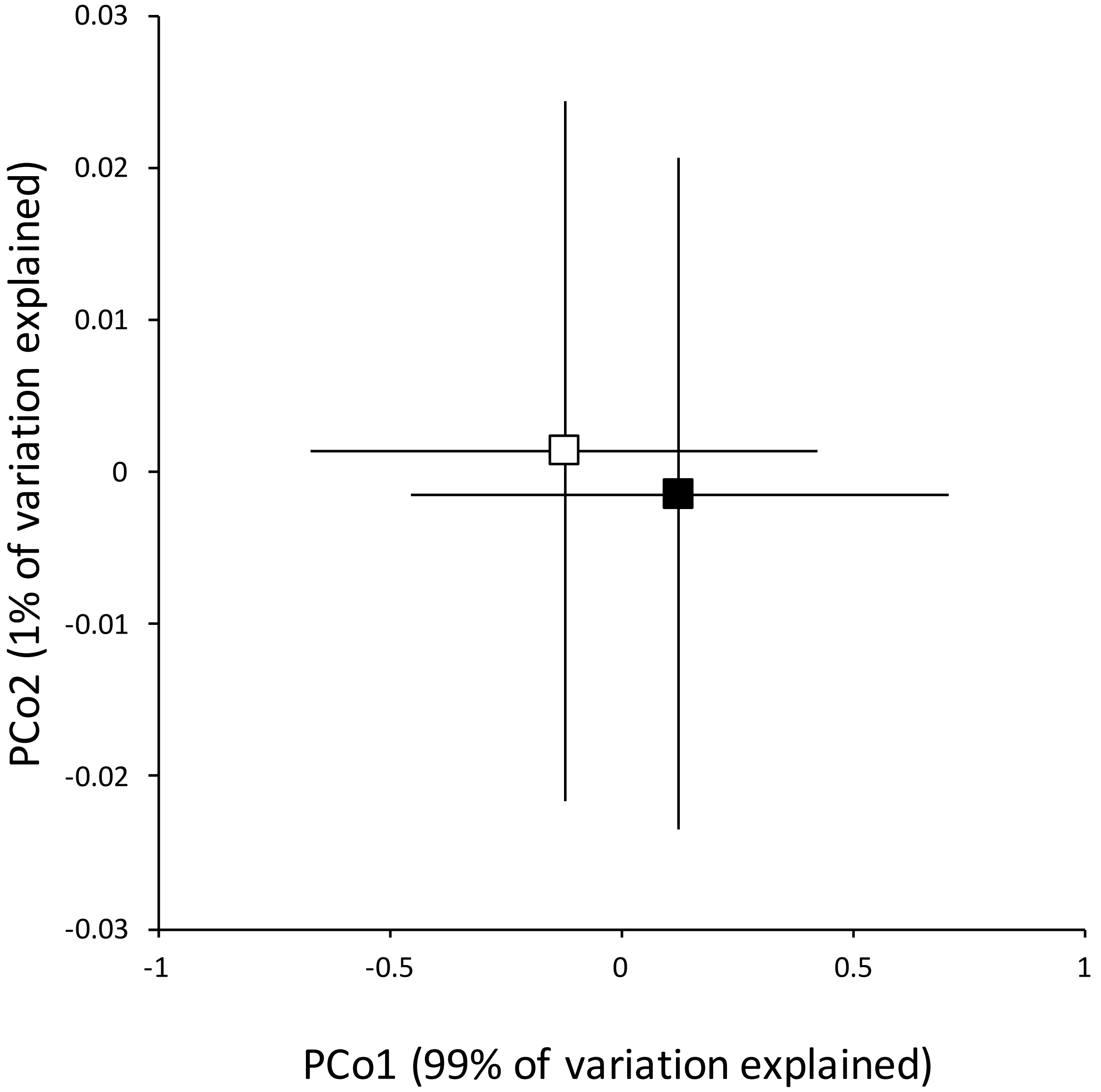

Supplemental Figure S3. The effect of experimental N deposition on the composition of SEED subsystem level 1 classifications. Ordinations were obtained from Principle Coordinates Analysis on based Euclidian distances of matR normalized data.
