# Supplementary material for: Anthropogenic N Deposition Slows Decay by Favoring Bacterial Metabolism: Insights from Metagenomic Analyses
Source: Front Microbiol. 2016 Mar 2;7:259. doi: 10.3389/fmicb.2016.00259 (PMC4773658; doi:10.3389/fmicb.2016.00259)
Supplement: Supplementary file 8 [file Image4.PDF]

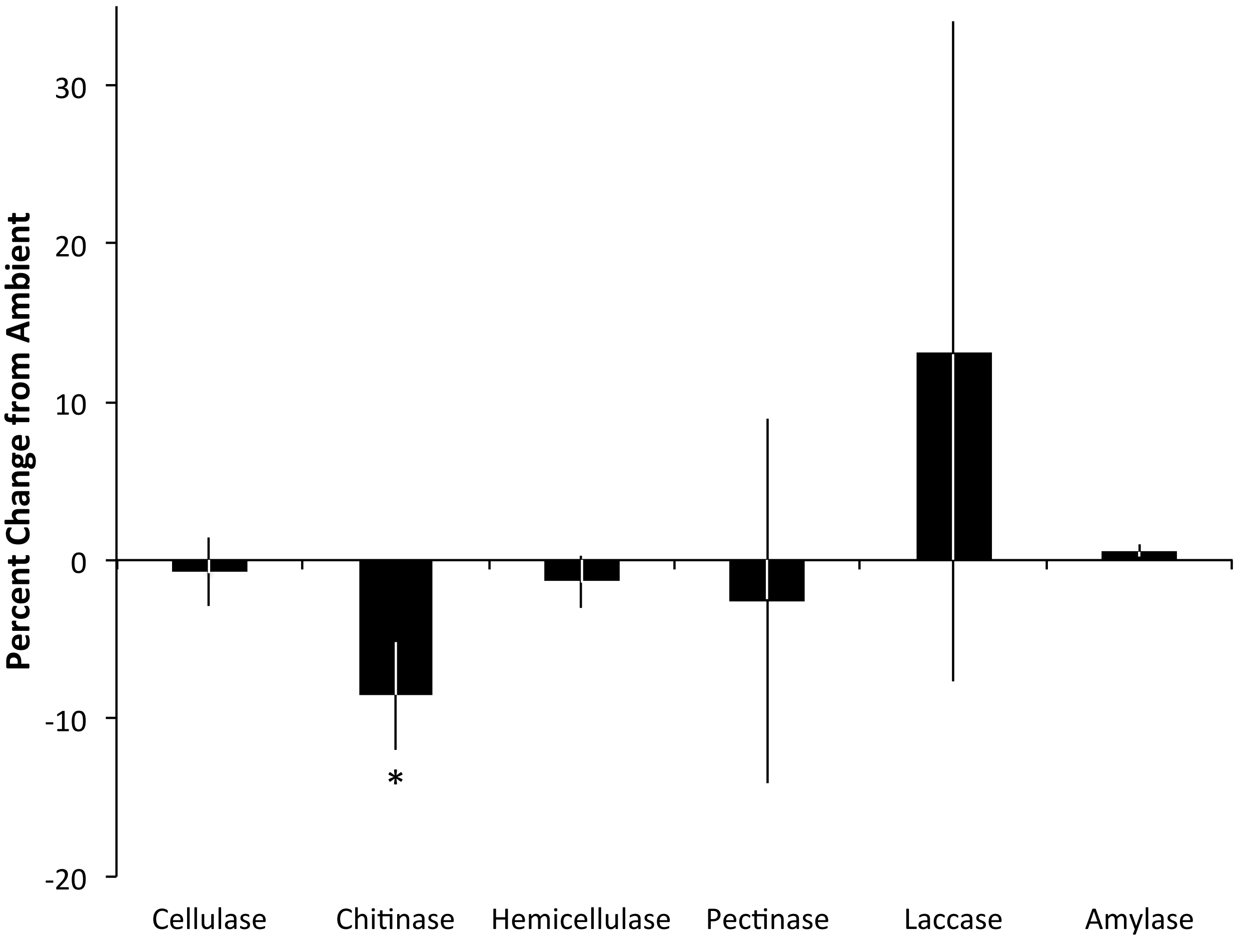

Supplemental Figure S4. The percent change from ambient of bacterial functional genes mediating the decay of plant litter compounds. Mean ± standard error (n = 12) values are presented.

* $P < 0.05$ by two-way ANOVA
