# Supplementary material for: Anthropogenic N Deposition Slows Decay by Favoring Bacterial Metabolism: Insights from Metagenomic Analyses
Source: Front Microbiol. 2016 Mar 2;7:259. doi: 10.3389/fmicb.2016.00259 (PMC4773658; doi:10.3389/fmicb.2016.00259)
Supplement: Supplementary file 9 [file Image5.PDF]

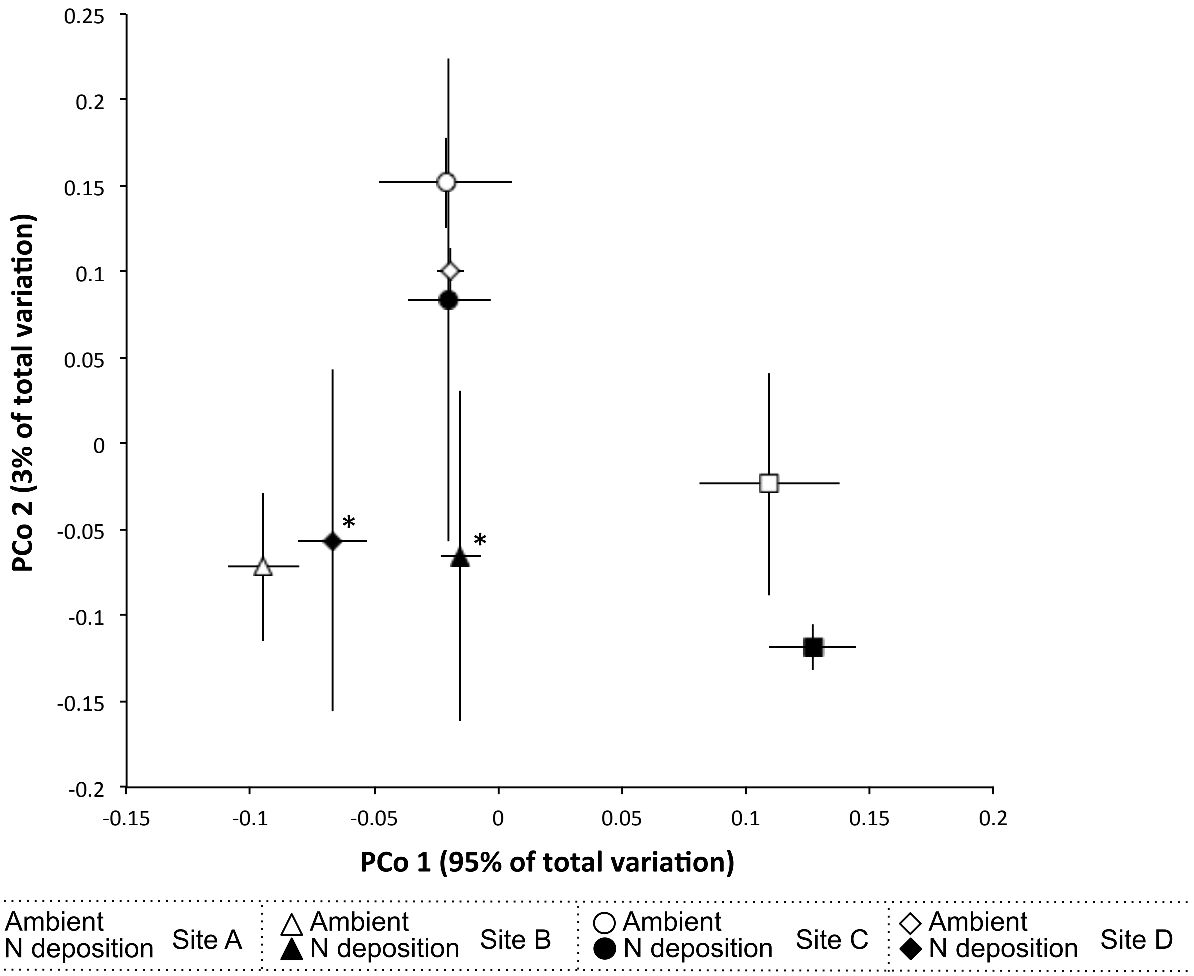

Supplemental Figure S5: The effect of experimental N deposition on the composition of bacterial functional genes mediating the decay of plant litter compounds. Mean ± standard error (n = 3) values are presented.

* $P < 0.05$ by pairwise PerMANOVA
